# Febuxostat Improves MASLD in Male Rats: Roles of XOR Inhibition and Associated JNK/NRF2/HO-1 Pathway Changes

**DOI:** 10.3390/ijms27021069

**Published:** 2026-01-21

**Authors:** Zhiyu Pu, Yangyang Cen, Bowen Yang, Kaijun Xing, Linxi Lian, Xi Chi, Jianjun Yang, Yannan Zhang

**Affiliations:** 1 Department of Nutrition and Food Hygiene, Faculty of Public Health, Ningxia Medical University, Yinchuan 750004, China; 2 Ningxia Key Laboratory of Environmental Factors and Chronic Disease Control, Yinchuan 750004, China

**Keywords:** MASLD, febuxostat, xanthine oxidoreductase, metabolomics, oxidative stress

## Abstract

Metabolic dysfunction-associated steatotic liver disease (MASLD) is a peril to public health. Xanthine oxidoreductase (XOR) is implicated in oxidative stress and lipid metabolism, which constitute the pathological basis of MASLD. As a specific XOR inhibitor, febuxostat therefore exhibits considerable potential for mitigating MASLD. However, the efficacy and underlying mechanisms of febuxostat in this context remain to be elucidated. Against this background, the present study aimed to observe the effect of febuxostat on the physiological changes of male MASLD rats and explore the related mechanisms. All rats were assigned to three groups: control, high-fat diet (HF), and high-fat diet with febuxostat (HF + F). After euthanasia, biosamples were immediately harvested to conduct an extensive suite of experiments, encompassing histological examination, assessment of biochemical and oxidative stress markers, serum non-targeted metabolomics, and Western blot analysis. Histological examination showed marked reductions in hepatic lipid accumulation and hepatocellular degeneration in the HF + F group relative to the HF group. Consistently, compared to the HF group, the HF + F group showed significant reductions in the elevated levels of plasma/hepatic lipids, and plasma oxidative stress markers (*p* < 0.05). Serum metabolomics revealed distinct metabolic profiles among groups, with 51 differential metabolites between HF + F and HF groups, with pathways such as taurine and hypotaurine metabolism and starch and sucrose metabolism being significantly altered (*p* < 0.05). Western blot analysis showed reduced *p*-JNK and increased NRF2 and HO-1 expression in the HF + F group (*p* < 0.05). In summary, we found that inhibiting XOR with febuxostat improved hepatic steatosis, serum metabolic dysregulation and systemic oxidative stress status, and it accompanied by JNK/NRF2/HO-1 pathway key molecule protein alterations in male MASLD rats.

## 1. Introduction

Metabolic dysfunction-associated steatotic liver disease (MASLD), a globally prevalent metabolic liver disorder, is tightly correlated with metabolic syndrome [1]. Hepatic injury serves as a core pathological feature of MASLD, which exhibits a progressive course from hepatic steatosis to liver fibrosis, cirrhosis, and eventually end-stage liver failure [2,3]. With the elevation of living standards and the growing prevalence of unhealthy lifestyles, the global prevalence of MASLD has surged, placing a substantial burden on healthcare systems worldwide [4]. However, the pathogenesis of MASLD remains incompletely understood, and no targeted pharmacotherapies have been approved for its clinical management to date. Accordingly, elucidating the underlying pathogenic mechanisms and developing targeted therapeutic strategies are imperative to alleviate the global healthcare burden, which constitutes a major priority in public health research.

Oxidative stress and lipid metabolism play a crucial role in the pathogenesis and progression of MASLD [5]. Oxidative stress induces lipid peroxidation [6] and inflammation [7], which sequentially accelerate the progression of hepatic steatosis and liver injury [8,9]. Lipid metabolic disorders, characterized by upregulated de novo lipogenesis, impaired fatty acid β-oxidation and dysregulated hepatic lipid transport, further exacerbate intrahepatic lipid accumulation [10]. Elevated oxidative stress and excessive lipid accumulation are commonly observed in MASLD patients [11]. It is well-documented that elevated oxidative stress initiates c-Jun N-terminal kinase (JNK) phosphorylation, which in turn represses nuclear factor erythroid 2-related factor 2 (NRF2)—a central oxidative stress regulator and upstream signaling molecule of Heme oxygenase-1 (HO-1)—thus constituting the JNK/NRF2/HO-1 pathway that mediates cellular antioxidant responses [12].

Xanthine oxidoreductase (XOR) is an enzyme involved in purine metabolism that generates reactive oxygen species (ROS) during catalysis, thus directly contributing to the development of oxidative stress [13]. Given that previous studies have demonstrated aberrant XOR overexpression and markedly increased oxidative stress in MASLD patients [14,15], XOR has emerged as a promising potential therapeutic target for MASLD. Febuxostat, a selective inhibitor of XOR, is widely used for treating hyperuricemia and gout due to its potent ability to lower serum uric acid levels [16]. However, whether febuxostat exerts a protective effect against MASLD, as well as the underlying molecular mechanisms mediating this effect, remain to be fully elucidated. Therefore, the present study was designed to investigate the effects of febuxostat on MASLD rats and explore the potential mechanisms involved.

To clarify the role of XOR inhibition in MASLD pathology and its mechanistic connection to metabolic regulation and the JNK/NRF2/HO-1 axis, we inhibited XOR activity in male MASLD rats using febuxostat. We then assessed its impacts on oxidative stress and lipid homeostasis and analyzed associated perturbations in metabolic profiles and XOR-related protein expression. We hope that this study will provide empirical evidence to inform future clinical management and therapeutic strategies for MASLD.

## 2. Results

### 2.1. Body Weight and Organ Coefficient (Liver and Epididymal)

**Figure 1:**
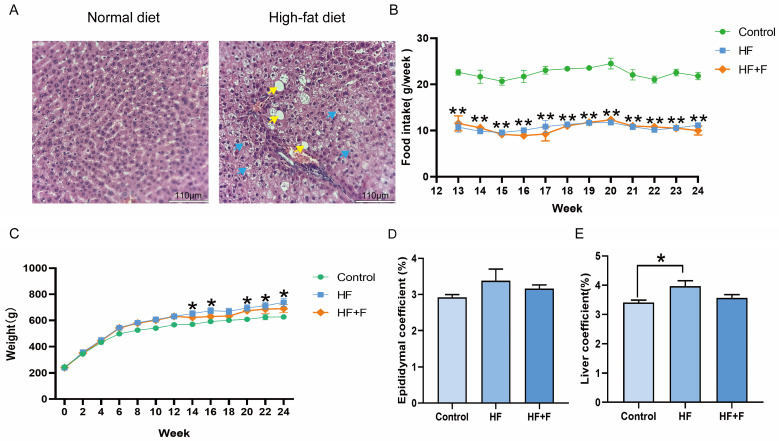
Liver histopathological changes in rats at the end of week 12 (**A**). *n* = 2 per group. Scale bar = 110 μm (10× objective). Yellow arrows indicate hepatic steatosis. Blue arrows indicate hepatocellular ballooning. Levels of (**B**) food intake, (**C**) body weight, (**D**) epididymal fat coefficient [calculated as (epididymal fat weight/body weight) × 100%] and (**E**) liver coefficient [calculated as (liver weight/body weight) × 100%] in different groups. For (**B**–**E**), *n* = 6 per group, Bars represent mean ± SEM, Statistical analyses were performed using unpaired one-way ANOVA followed by Tukey’s post hoc test, * *p* < 0.05, ** *p* < 0.01, HF group versus Control group.

Histopathological analysis revealed prominent hepatocellular ballooning and hepatic steatosis in rats fed a high-fat diet (HF) at the end of week 12 (Figure 1A), whereas no such abnormalities were observed in the normal diet control group. These findings confirmed the successful induction of the MASLD model. Following the initiation of febuxostat intervention at this time point, weekly food intake was monitored. As shown in Figure 1B, no significant difference in weekly food intake was found between the HF group and the HF + febuxostat (HF + F) group (*p* > 0.05).

As shown in Figure 1C, the body weights of the three groups were approximately equivalent at the initiation of the experiment. Following prolonged feeding with different diets, the HF group exhibited significantly higher body weight than the control group from week 14 onwards (*p* < 0.05). In contrast, the HF + F group showed no significant difference in body weight compared with either the HF group or the control group throughout the experimental period. Consistent with the body weight findings, the HF + F group showed no significant reduction in the epididymal fat coefficient (Figure 1D) or liver coefficient (Figure 1E) compared with the HF group. The data corresponding to Figure 1D,E are presented as mean ± standard error of the mean (SEM) in Table S1.

### 2.2. Histological Analysis 

**Figure 2:**
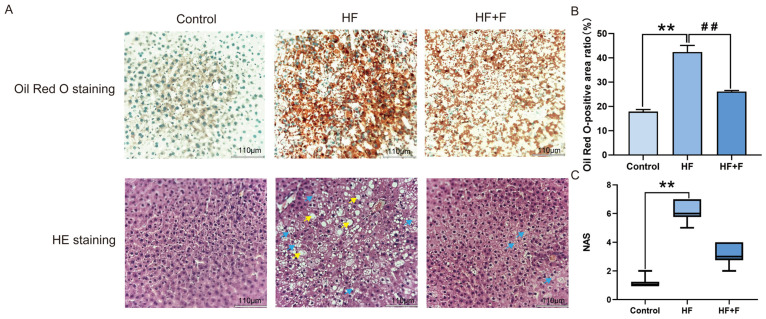
Hepatic histopathological analysis. Liver histology (**A**): Oil Red O staining (lipid droplets are stained orange-red; nuclei are stained blue-green). HE staining (nuclei are stained blue-purple; cytoplasm, extracellular matrix, and other eosinophilic structures are stained pink). Scale bar = 110 μm (10× objective). Yellow arrows indicate hepatic steatosis. Blue arrows indicate hepatocellular ballooning. Oil Red O-positive area ratio (**B**). Bars represent mean ± SEM. *n* = 3 per group. Statistical analyses were performed using one-way ANOVA followed by Tukey’s post hoc test. ** *p* < 0.01. ^##^ *p* < 0.01. NAS (**C**). Bars represent median and interquartile range (IQR). *n* = 3 per group. Statistical analyses were performed using Kruskal–Wallis test followed by Dunn’s multiple comparisons. ** *p* < 0.01.

Histopathological analysis of liver tissues revealed distinct patterns across the three groups (Figure 2A, Figure S1 and Figure S2). Oil Red O staining indicated that the HF group had the most severe intracellular lipid deposition. Consistently, Hematoxylin and eosin (HE) staining showed prominent hepatocellular vacuolar degeneration in the same group. In contrast, the HF + F group exhibited a significantly reduced Oil Red O-positive area (*p* < 0.01, Figure 2B) compared to the HF group. As shown in Figure 2C, no significant difference was demonstrated in the NAFLD Activity Score (NAS) between the HF group and the HF + F group. Consistent with the overall NAS results, the HF + F group also exhibited no significant reduction in the individual scores of steatosis, lobular inflammation, or hepatocyte ballooning compared with the HF group (see detailed grading criteria in Table 1).

### 2.3. Biochemical Indicator Analysis 

**Figure 3:**
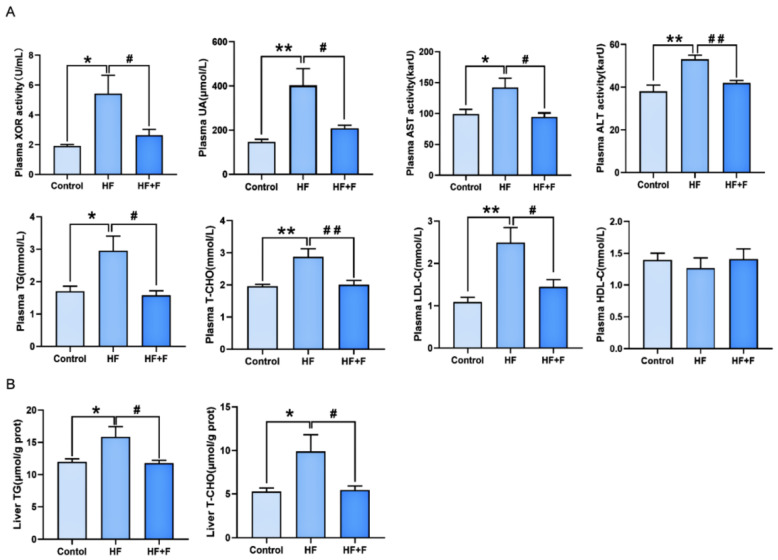
Biochemical analysis in plasma (**A**) or liver (**B**). *n* = 6 per group. Bars represent mean ± SEM. Statistical analyses were performed using one-way ANOVA followed by Tukey’s post hoc test. * *p* < 0.05. ** *p* < 0.01. ^#^ *p* < 0.05. ^##^ *p* < 0.01.

Plasma biochemical analysis (Figure 3A) showed that the HF group had significantly elevated levels of XOR activity, uric acid (UA), triglycerides (TG), total cholesterol (T-CHO), low-density lipoprotein cholesterol (LDL-C), aspartate aminotransferase (AST), and alanine aminotransferase (ALT) compared to the control group (all *p* < 0.05). These elevated indicators were all significantly reduced in the HF + F group relative to the HF group (*p* < 0.05). Consistent with this trend, liver biochemical analysis (Figure 3B) further revealed that the HF + F group exhibited significantly lower hepatic levels of TG (*p* < 0.05) and T-CHO (*p* < 0.05) than the HF group.

### 2.4. Oxidative Stress Parameters

**Figure 4:**
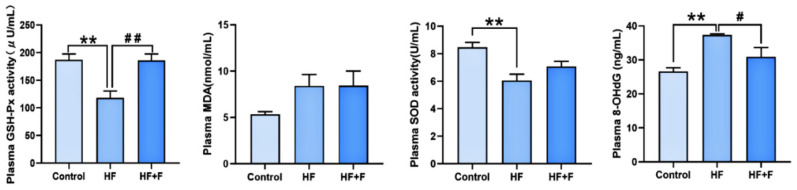
Oxidative stress in plasma. Plasma levels of GSH-Px activity, malondialdehyde (MDA), SOD activity and 8-OHdG in different groups. *n* = 6 per group. Bars represent mean ± SEM. Statistical analyses were performed using one-way ANOVA followed by Tukey’s post hoc test. ** *p* < 0.01. ^#^ *p* < 0.05. ^##^ *p* < 0.01.

The plasma oxidative stress parameters among the groups were illustrated in Figure 4. Compared with the control group, plasma glutathione peroxidase (GSH-Px) activity (*p* < 0.01) and plasma superoxide dismutase (SOD) activity (*p* < 0.01) were notably decreased in the HF group, while plasma 8-hydroxy-2’-deoxyguanosine (8-OHdG) was markedly increased (*p* < 0.01). These changes were significantly reversed by febuxostat treatment: relative to the HF group, the HF + F group exhibited higher plasma GSH-Px activity (*p* < 0.01) and a lower 8-OHdG level (*p* < 0.05).

### 2.5. Serum Non-Targeted Metabolomics Results

Using untargeted metabolomics, we examined the serum metabolites by ultra-high performance liquid chromatography-tandem mass (UHPLC-MS/MS) in both positive and negative ion modes. A total of 13,149 features were obtained. The workflow for data acquisition in both ionization modes is depicted in the Supplementary Material (Figure S3).

**Figure 5:**
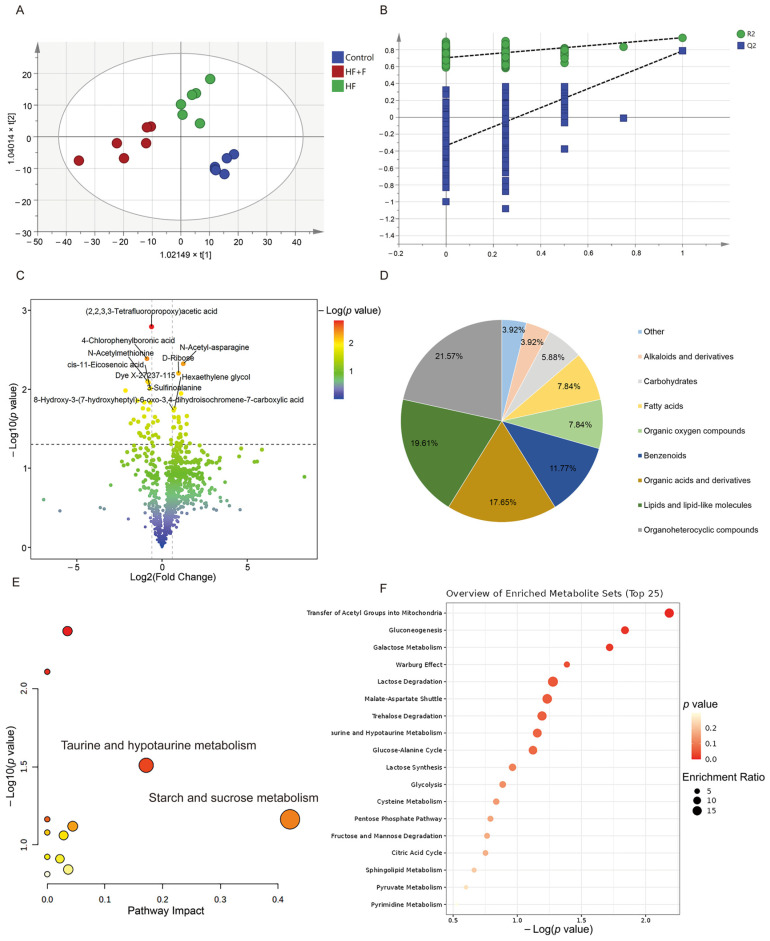
Serum non-targeted metabolomics results. OPLS-DA analysis score chart (**A**) and OPLS-DA model validation (**B**) among three groups. A volcano plot (**C**), including 2, 2, 3, 3-Tetrafluoropropoxy)acetic acid, 4-Chlorophenylboronic acid, N-Acetyl-asparagine, 3-Sulfinoalanine, N-Acetylmethionine, Dye X-27237-115, cis-11-Eicosenoic acid, Anacardic acid, 8-Hydroxy-3-(7-hydroxyheptyl)-6-oxo-3, 4, dihydroisochromene-7-carboxylic acid, D-Ribose, and so on. A classification pie chart (**D**) between HF + F group and HF group. Metabolic pathway analysis (**E**) of 51 differential metabolites. Color coding: Dot color scales with −log10 (*p* value), where redder dots denote higher significance and whiter dots denote lower. Enrichment analysis (**F**) of 51 differential metabolites. *n* = 6 per group.

The orthogonal partial least squares—discriminant analysis (OPLS-DA) score plots (Figure 5A) delineated a distinct pattern of segregation amongst the tripartite groups, thereby indicating that the metabolic phenotypes characteristic of each group were markedly disparate. Furthermore, the overfitting verification results (Figure 5B) demonstrated that the regression line did not exhibit characteristics of overfitting (Figure 5B, R^2^ = 0. 719, Q^2^ = 0.178), suggesting an appropriate model fit.

Subsequently, based on metabolomic findings (Table S2), in total, 51 metabolites exhibited distinguishable discrepancies between the HF + F and the HF group. Next, a volcano plot revealed that, the HF + F group exhibited a significant up-regulation of 27 differential metabolites, whereas conversely, 24 differential metabolites were markedly down-regulated (Figure 5C). The identified altered metabolites encompassed a variety of classes, such as organoheterocyclic compounds (e.g., 1-(2-Hydroxyethyl)pyrazole, 2, 4, 6-Trimethylpyridine), lipids and lipid-like molecules (e.g., cis-7-Hexadecenoic acid, cis-Pinonic acid), organic acids and derivatives (e.g., 3-Sulfinoalanine, Pro-Gly), benzenoids (e.g., Anacardic acid), fatty acids (e.g., 2-Hydroxyhexanedioic acid) and among others (Figure 5D).

Significantly, as shown in the metabolic pathway analysis results (Figure 5E), 12 metabolic pathways were perturbed within the HF + F group. Among them, the metabolism of taurine and hypotaurine, as well as starch and sucrose metabolism, were the most significantly disrupted pathways (impact value > 0.15). Moreover, the enrichment analysis depicted a total of 18 metabolic pathways, including transfer of acetyl groups into mitochondria, gluconeogenesis, galactose metabolism and so on (Figure 5F).

### 2.6. Protein Expression Within the JNK/NRF2/HO-1 Pathway

**Figure 6:**
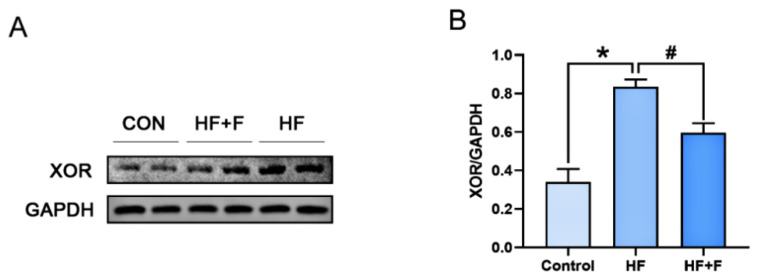
The protein expression of XOR in liver. (**A**) Representative immunoblot of proteins of the XOR protein. Relative expression of (**B**) XOR normalized to Glyceraldehyde-3-Phosphate Dehydrogenase (GAPDH). *n* = 6 per group. Bars represent mean ± SEM. Statistical analyses were performed using one-way ANOVA followed by Tukey’s post hoc test. * *p* < 0.05. ^#^ *p* < 0.05.

**Figure 7:**
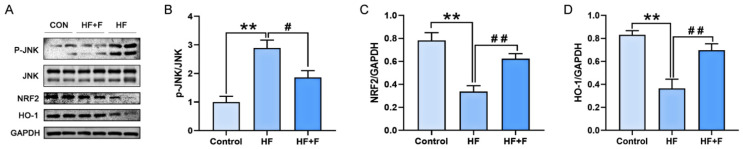
The protein expression of JNK/NRF2/HO-1 signaling pathway in liver. (**A**) Representative immunoblot of proteins of the JNK/NRF2/HO-1 pathway. Relative expression of (**B**) *p*-JNK normalized to JNK. (**C**) NRF2 normalized to GAPDH. (**D**) HO-1 normalized to GAPDH. *n* = 6 per group. Bars represent mean ± SEM. Statistical analyses were performed using one-way ANOVA followed by Tukey’s post hoc test. ** *p* < 0.01. ^#^ *p* < 0.05. ^##^ *p* < 0.01.

As demonstrated by Western blot (WB) analysis (Figure 6), hepatic XOR protein expression was significantly down-regulated in the HF + F group compared with the HF group (*p* < 0.05). To further elucidate the molecular mechanisms underlying the effects of febuxostat against MASLD, additional WB analysis (Figure 7) revealed that the hepatic phosphorylated c-Jun N-terminal kinase (*p*-JNK) protein expression ratio was notably decreased in the HF + F group relative to the HF group (*p* < 0.05). Conversely, marked upregulation of the expression of NRF2 and HO-1—two downstream proteins—was observed (*p* < 0.01).

## 3. Discussion

MASLD, which is typified by increased oxidative stress and lipid accumulation [17,18], is the most common multisystem clinical disease worldwide [19]. Despite recent significant progress in understanding the pathogenesis of MASLD [20], the specific role of XOR in this disease remains incompletely understood. Accordingly, in the present study, we found that inhibition of XOR by febuxostat alleviates pathological manifestations in a MASLD rat model, including hepatic lipid accumulation, serum metabolic disorders, and systemic oxidative stress, and this effect coincides with reduced JNK phosphorylation and enhanced NRF2 and HO-1 protein expression.

### 3.1. Febuxostat Ameliorates MASLD Core Pathologies Through XOR Inhibition

XOR, a key enzyme in purine metabolism, is targeted by the well-characterized inhibitor febuxostat, which exerts therapeutic effects on MASLD [21,22]. In our experiment, febuxostat significantly reduced XOR protein expression and activity as well as its metabolic product, UA. Oil Red O staining results demonstrated that febuxostat-mediated XOR inhibition significantly ameliorated hepatic lipid deposition in male high-fat diet-induced MASLD rats. This ameliorative effect was further supported by pronounced reductions in hepatic TG and T-CHO levels. This observation is consistent with a recent clinical study, which reported that XOR inhibitors alleviate hepatic lipid accumulation in patients with mild MASLD [23]. Moreover, blood biochemical analyses demonstrated that febuxostat-mediated XOR inhibition substantially rectified high-fat diet-induced systemic lipid dysregulation in male MASLD rats. This observation is further supported by prior work showing that febuxostat improves insulin resistance and lipid peroxidation—key drivers of aberrant lipid metabolism [24]. In contrast, no significant differences were observed in individual scores of steatosis, lobular inflammation, or hepatocyte ballooning, which may be attributed to the fact that our high-fat diet-induced MASLD model only developed mild-to-moderate hepatic steatosis without progressing to steatohepatitis—thus limiting the additional observable therapeutic efficacy of febuxostat. Collectively, these results reinforce the notion that inhibiting XOR with febuxostat exerts a potential therapeutic effect on high-fat diet-induced MASLD by ameliorating lipid dysregulation in rat models.

Notably, while febuxostat effectively suppressed XOR enzymatic activity in our model, we also observed a concomitant reduction in XOR protein expression. This finding extends beyond the established view of febuxostat as a purely enzymatic inhibitor. The decrease in XOR protein likely results from an indirect feedback mechanism: XOR expression is known to be upregulated by a variety of factors such as inflammatory cytokines and ROS [25]. By inhibiting XOR activity, febuxostat reduces the production of ROS and uric acid (UA), both of which can transcriptionally enhance XOR gene expression via the p38 MAPK/Egr-1 signaling axis [26]. Thus, febuxostat disrupts this self-amplifying loop, leading to decreased XOR protein synthesis. Together, these results confirm the efficacy of febuxostat and provide new mechanistic insight: it regulates XOR through dual modes—inhibiting its enzymatic activity and reducing its protein abundance. Further studies are needed to fully clarify this regulatory pathway in MASLD.

### 3.2. Inhibiting XOR with Febuxostat Reprograms Metabolism Pathways in Male MASLD Rats

In the present study, metabolic pathway analysis demonstrated that inhibition of XOR with febuxostat was implicated in several key metabolic pathways, including taurine and hypotaurine metabolism, starch and sucrose metabolism, and transfer of acetyl groups into mitochondria. These pathways are underscoring the broader metabolic regulatory potential of febuxostat that extends beyond its role in ameliorating lipid dysregulation.

Among these pathways, the taurine and hypotaurine metabolism pathway is a key branch of sulfur-containing amino acid metabolism and a core defensive mechanism against oxidative damage [27]. Notably, metabolomic analysis revealed that febuxostat significantly elevated levels of 3-sulfinoalanine, a critical intermediate in this pathway, in male MASLD rats. This finding suggests a feedback regulatory mechanism underlying the pathway’s antioxidant activity: On one hand, 3-sulfinoalanine itself is involved in ROS scavenging [28]; on the other hand, its accumulation indirectly modulates the biosynthetic efficiency of hypotaurine and taurine, thereby regulating the pathway’s overall antioxidant capacity to adapt to febuxostat-induced alterations in the in vivo redox microenvironment [29].

Moreover, starch and sucrose metabolism serves as the primary pathway for carbohydrate catabolism, supplying glucose as the core substrate for energy metabolism [30]. Under XOR inhibition, glucose was identified as a pathway-associated differential metabolite in MASLD rats. According to existing literature, inhibition of ROS generation enhances glucose uptake and utilization in adipocytes and hepatocytes, while simultaneously mitigating excessive glucose release from aberrant hepatic glycogenolysis [31]. These dual regulatory effects correct the imbalance in glucose homeostasis, thereby laying the foundation for the subsequent attenuation of lipid dysregulation—findings that align with our study results.

As for transfer of acetyl groups into mitochondria, it is the rate-limiting step linking glycolysis, fatty acid β-oxidation, and the tricarboxylic acid (TCA) cycle, mediated by the citrate-malate shuttle [32,33]. Cytosolic acetyl-CoA combines with oxaloacetate to form citrate, which enters mitochondria and is cleaved to regenerate acetyl-CoA for oxidative phosphorylation (OXPHOS). Accumulating evidence indicates that ROS disrupt this shuttle by oxidizing mitochondrial citrate synthase (CS), reducing its catalytic activity and impairing acetyl-CoA mitochondrial entry [34,35]. Resultant cytosolic acetyl-CoA accumulation serves as a substrate for de novo fatty acid synthesis, exacerbating lipid dysregulation. Consistent with these observations, our analytical results demonstrate that febuxostat improves the acetyl group transfer pathway in mitochondria of MASLD rats, suggesting that XOR inhibition with febuxostat restores transfer efficiency via preserving mitochondrial function. To summarize, all analytical findings collectively suggest that metabolic pathway reprogramming is induced in male rats with MASLD via inhibition of XOR with febuxostat.

### 3.3. XOR Inhibition by Febuxostat Attenuates Systemic Oxidative Stress: Concurrent with JNK Down-Regulation and NRF2/HO-1 Up-Regulation

Oxidative stress is defined as an imbalance between ROS production and antioxidant capacity. Mounting evidence confirms that high-fat diets upregulate XOR activity, promoting excessive ROS and UA generation to exacerbate oxidative stress in MASLD pathogenesis [36,37,38]. Notably, UA-induced hepatic injury is closely linked to oxidative stress: UA enhances ROS production via NADPH oxidase activation and mitochondrial dysfunction [39], while ROS impairs renal UA excretion to form a vicious cycle during MASLD progression [40]. In contrast, febuxostat-mediated XOR inhibition disrupts this cycle by suppressing XOR activity to reduce ROS, alleviating oxidative stress-induced lipid peroxidation and hepatocellular damage. Concomitantly, reduced UA mitigates UA-driven inflammation and de novo lipogenesis, further decreasing ROS substrates (e.g., free fatty acids). Furthermore, recent evidence has demonstrated that excessive XOR activity, localized in both intestinal epithelial cells and gut microbes, also contributes to ROS overproduction by disrupting intestinal barrier integrity and facilitating lipopolysaccharide (LPS) translocation [41,42]. Collectively, these findings are consistent with our results, demonstrating that febuxostat ameliorates systemic oxidative stress in male MASLD rats.

NRF2, a key transcription factor modulated by JNK phosphorylation, mediates antioxidant responses to oxidative stress via the activation of downstream target genes including HO-1, which is a core component of the JNK/NRF2/HO-1 signaling pathway. In our study, alleviation of plasma oxidative stress in MASLD rats by febuxostat-mediated XOR inhibition was accompanied by decreased JNK phosphorylation and elevated NRF2 and HO-1 protein expression—indicating that these phenotypic improvements are accompanied by alterations in the JNK/NRF2/HO-1 signaling pathway. Notably, the JNK/NRF2/HO-1 pathway is closely linked to energy metabolism [43,44,45], For instance, accumulating data demonstrate that NRF2 activation enhances mitochondrial oxidative phosphorylation and ATP synthesis [46], implying that NRF2 may facilitate the mitochondrial translocation of acetyl groups—a critical substrate of the tricarboxylic acid cycle—via optimizing mitochondrial function. Similarly, HO-1 protects the activity of key glycolytic enzymes such as hexokinase and glycogen phosphorylase by reducing ROS levels, thereby alleviating glucotoxicity-induced damage [44]. Additionally, relevant studies have reported that NRF2 modulates taurine and hypotaurine metabolism through regulating oxidative stress [47], thereby maintaining metabolic homeostasis. These findings align with our results, indicating that the JNK/NRF2/HO-1 pathway may act as a critical mediator linking febuxostat-induced XOR inhibition to improved metabolic homeostasis in MASLD.

### 3.4. Potential Intestinal Mechanisms Contributing to Febuxostat’s Efficacy

Beyond its direct hepatic effects, our findings, along with emerging literature, prompt consideration of the intestine as a potential site of action for febuxostat in MASLD. As noted, XOR activity in intestinal epithelial cells and gut microbiota contributes to ROS overproduction and compromises barrier function, facilitating the translocation of pro-inflammatory agents like LPS into the portal circulation—a key driver of hepatic inflammation and steatosis in MASLD [48]. We speculate that febuxostat-mediated XOR inhibition may also target this intestinal compartment. By reducing XOR-derived ROS and UA in the gut, febuxostat could potentially (1) reinforce intestinal barrier integrity, reducing systemic exposure to gut-derived endotoxins [49]; (2) modulate the gut microbial ecosystem, as XOR activity influences the local redox environment crucial for microbial composition [2,50]; and (3) alter the profile of gut-derived metabolites (e.g., bile acids, short-chain fatty acids) that are known regulators of hepatic metabolism and inflammation [51]. This proposed “gut-liver axis” mechanism would operate in concert with the observed systemic metabolic reprogramming and attenuation of plasma oxidative stress. Future studies directly assessing intestinal XOR activity, barrier markers, microbiome, and fecal metabolome in febuxostat-treated MASLD models are warranted to validate this hypothesis and elucidate its relative contribution to the drug’s overall therapeutic benefit.

### 3.5. Strengths and Limitations

This study has several key strengths. We employed a multi-level approach integrating histology, biochemistry, metabolomics, and molecular analysis, offering a comprehensive assessment of febuxostat’s effects in MASLD. The application of non-targeted metabolomics was particularly valuable, revealing 51 altered metabolites and identifying key reprogrammed metabolic pathways. We also provide novel evidence linking XOR inhibition to modulation of the JNK/NRF2/HO-1 pathway, offering a mechanistic explanation for the observed reduction in systemic oxidative stress. Importantly, by focusing on the repurposing potential of the clinically approved drug febuxostat, our findings hold direct translational relevance for a condition lacking effective therapies.

However, certain limitations warrant consideration. First, the exclusive use of male rats, chosen for their higher susceptibility to diet-induced MASLD [52], implies our findings may be sex-specific. Future studies should incorporate female models to elucidate sex-dependent mechanisms. Second, oxidative stress and metabolomic analyses were confined to serum; validating key metabolite alterations in liver tissue is needed to accurately reflect tissue-specific perturbations. Third, the failure to determine serum non-esterified fatty acids (NEFAs) represents a limitation, as this parameter is important for linking systemic lipid dysregulation to hepatic steatosis [53]. Additionally, the internal reference protein GAPDH may be unstable in liver steatosis models; subsequent research will employ more stable housekeeping proteins to enhance reliability. Finally, to establish causality, future in vitro studies will involve supplementation or depletion of the identified key metabolites to directly assess their impact on the JNK/NRF2/HO-1 pathway.

## 4. Materials and Methods

### 4.1. Experimental Design

**Figure 8:**
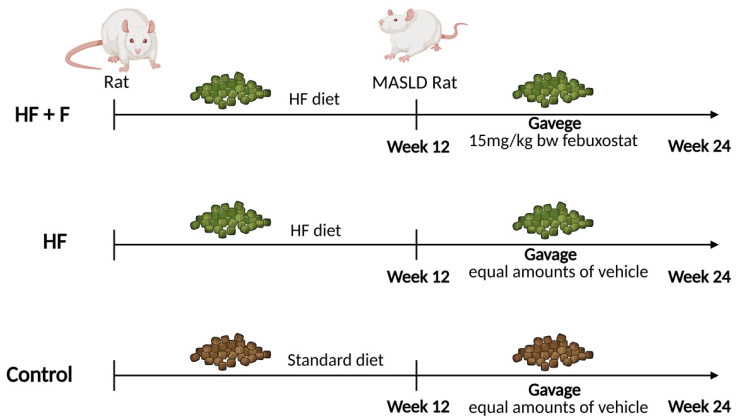
Flow chart explaining experimental design of the present study. Created in BioRender. P, Z. (2025) https://BioRender.com/p80i976 (accessed on 12 January 2026).

Twenty-four male Sprague–Dawley (SD) rats (6–8 weeks old, 200 ± 20 g) were obtained from the Experimental Animal Center of Ningxia Medical University (IACUC license number: NYLAC-2021-192). The rats were housed in a specific-pathogen-free (SPF) environment maintained at 22 ± 1 °C with 50% relative humidity and a 12 h light/12 h dark cycle. Based on their initial body weights, the rats were randomly allocated to three experimental groups, as outlined in the experimental design (Figure 8). Specifically, these groups included a control group (Control, *n* = 8) fed a standard rodent chow (Xietong Pharmaceutical Science and Engineering Co., Ltd., Beijing, China); a high-fat diet group (HF, *n* = 8); and a high-fat diet + febuxostat group (HF + F, *n* = 8). The latter two groups were administered a purified high-fat rodent chow (MD12033, Medicience Ltd., Yangzhou, China). The detailed macronutrient composition (carbohydrates, lipids, and proteins) of all experimental diets is provided in the Supplementary Materials (Supplementary S5). Throughout the entire experimental period, all rats had free access to food and water (with no modifications to the dietary composition or source) and were weighed weekly.

Notably, two rats were randomly selected from each diet group (normal diet group and high-fat diet group) and humanely euthanized on the 12th weekend. NAS, a scoring system originally and widely validated for the pathological assessment of non-alcoholic fatty liver disease (NAFLD)—now reclassified as MASLD—was used to quantify the severity of liver injury and thereby confirm the successful establishment of MASLD models. Following the successful induction of MASLD via 12 weeks of high-fat diet feeding, rats in the HF + F group were administered febuxostat tablets (15 mg/kg body weight, Jiangsu Wanbang Biochemical Pharmaceutical Group Co., Ltd., Yangzhou, China) via intragastric gavage for an additional 12 weeks. In parallel, rats in the Control and HF groups received an equivalent volume of distilled water (the vehicle for febuxostat tablets) via the same route of administration. Upon completion of the experiment, all rats were subjected to a 12 h fasting period and then anesthetized with isoflurane inhalation for sustained anesthesia. While fully anesthetized and unresponsive, blood was collected via the abdominal aorta, leading to exsanguination and death. Following confirmation of death, target tissues including epididymal fat and liver were harvested and weighed. Among these tissues, a portion of the liver was fixed for histopathological analysis, while the remaining portion was snap-frozen in liquid nitrogen and subsequently stored at −80 °C for further analysis.

All experimental protocols were meticulously carried out in adherence to the ARRIVE guidelines, as well as the U. S. Public Health Service Policy on the Humane Care and Use of Laboratory Animals.

### 4.2. Histological Examination

For HE staining, a random segment was excised from each liver sample that had been fixed and stored in 4% paraformaldehyde (Tianjin Damao Chemical Reagent Factory, Tianjin, China). The liver tissues were then embedded in paraffin and sectioned into routine paraffin sections (5 μm) using a RM2016 microtome (Shanghai Leica Instrument Co., Ltd., Shanghai, China). Following deparaffinization and hydration, the sections were stained with hematoxylin-eosin (Nanjing Jiancheng Institute of Bioengineering, Inc., Nanjing, China), followed by differentiation, dehydration, and mounting. The severity of hepatocyte degeneration was evaluated based on these stained sections.

For Oil Red O staining, a random segment was excised from each liquid nitrogen-frozen liver sample. The tissues were subsequently embedded in optimal cutting temperature (OCT) medium and sectioned into 4 μm-thick frozen sections using a CryoStar NX50 cryostat (Thermo Scientific, Waltham, MA, USA). The sections were stained with Oil Red O solution (Nanjing Jiancheng Institute of Bioengineering, Nanjing, China), followed by brief differentiation and hematoxylin counterstaining to evaluate the severity of fat accumulation.

### 4.3. Biochemical Indicators Measurement

Blood was collected from each male rat into Ethylenediaminetetraacetic acid (EDTA)-anticoagulant tubes, with all samples analyzed individually without pooling. The samples were then centrifuged at 3000 rpm and 4 °C to separate plasma. Plasma levels of high-density lipoprotein cholesterol (HDL-C), LDL-C, T-CHO, TG, UA, as well as the activities of AST, ALT, and XOR, were determined by enzymatic colorimetry according to the manufacturer’s instructions for the test kits (Nanjing Jiancheng Institute of Bioengineering, Inc., Nanjing, China). Additionally, after sample pretreatment per the manufacturer’s instructions, hepatic levels of TG and T-CHO were quantified using the identical standardized protocol described previously.

### 4.4. Oxidative Stress Indicators Measurement

Plasma SOD activity was determined by colorimetry according to the manufacturer’s instructions for the test kit (Solarbio Biotechnology Co., Ltd., Beijing, China). Similarly, plasma GSH-Px activity was measured via the 5,5’-dithiobis (2-nitrobenzoic acid) (DTNB) method following the protocol provided by Beyotime Biotechnology Co., Ltd. (Beijing, China). Additionally, plasma MDA levels were quantified using the thiobarbituric acid (TBA) assay, in accordance with the instructions of the test kit from Nanjing Jiancheng Institute of Bioengineering, Inc. (Nanjing, China). Plasma 8-OHdG concentrations were determined by enzyme-linked immunosorbent assay (ELISA) using commercial kits from Elabscience Biotechnology Co., Ltd. (Wuhan, China).

### 4.5. Serum Non-Targeted Metabolomics

The methodologies employed for non-targeted metabolomics were modified and refined from prior studies [54,55,56]. The detailed procedures are as follows:

Sample preparation: Briefly, 100 μL of serum was transferred into an Eppendorf tube (EP tube), and 400 μL of extraction solvent (methanol: acetonitrile, 1:1, *v*/*v*) was added. The mixture was vortexed for 30 s, sonicated in an ice-water bath for 10 min, and then incubated at −40 °C for 1 h to achieve complete protein precipitation. Finally, the samples were centrifuged at 12,000× *g* for 15 min at 4 °C, and the supernatants were collected for subsequent analysis.

Quality control (QC): To evaluate the system stability and reproducibility of the analytical process, a pooled QC sample was prepared prior to instrumental analysis by mixing equal volumes of supernatants from all individual samples. This QC sample was injected repeatedly throughout the liquid chromatography–tandem mass spectrometry (LC–MS/MS) run sequence to monitor instrument performance and data consistency.

LC–MS/MS analysis: Chromatographic separation was conducted on a Vanquish ultra-high-performance liquid chromatography (UHPLC) system (Thermo Fisher Scientific, Waltham, MA, USA) equipped with a Waters ACQUITY UPLC BEH Amide column (2.1 × 50 mm, 1.7 µm). The flow rate was set at 0.3 mL/min, and the injection volume was 2 µL. The mobile phase comprised two components: (A) 25 mM ammonium acetate supplemented with 25 mM ammonia in water, and (B) acetonitrile. The eluates were introduced into an Orbitrap Exploris 120 mass spectrometer (Thermo Fisher Scientific, Waltham, MA, USA) controlled by Xcalibur 4.4 software (Thermo Fisher Scientific, Waltham, MA, USA). Key operating parameters were configured as follows: sheath gas flow, 50 arbitrary units (arb); auxiliary gas flow, 15 arb; capillary temperature, 320 °C full-scan resolution, 60,000; MS/MS resolution, 15,000; stepped normalized collision energy (NCE), 20/30/40; spray voltage, +3.8 kV (positive ion mode) or −3.4 kV (negative ion mode).

Data processing: Raw files were converted to mzXML format using ProteoWizard 3.0 software. Peak picking and alignment were performed with an in-house developed R package (v4.2.2), yielding a feature table containing retention time and m/z values. Features detected in less than 80% of the samples were excluded, and the remaining data matrix was normalized to the total peak area. Metabolite annotation was conducted against the BiotreeDB v2.1 database using MS/MS spectral data. Only hits with a similarity score ≥ 0.3 were retained. The data analysis was conducted using the internal standard normalization method to ensure accuracy and consistency.

### 4.6. Western Blot

Liver tissue (50 mg) from each rat was homogenized on ice in lysis buffer supplemented with protease and phosphatase inhibitors (Jiangsu KeyGEN Biotechnology Co., Ltd., Nanjing, China) to maintain protein integrity. Individual samples were analyzed without pooling, and the protein concentration in each lysate was quantified via a BCA protein assay kit (Jiangsu KeyGEN Biotechnology Co., Ltd., Nanjing, China).

Each rat protein sample was electrophoresed on 7.5% or 10% sodium dodecyl sulfate-polyacrylamide gel electrophoresis (SDS-PAGE) gels and then transferred to polyvinylidene fluoride (PVDF) membranes. The membranes were blocked with a 5% nonfat milk solution, followed by incubation overnight at 4 °C with the following primary antibodies: XOR mouse mAb (0.1% concentration, sc-398548, Santa Cruz Biotechnology, Inc., Dallas, TX, USA), JNK mouse mAb (0.1% concentration, sc-7345, Santa Cruz Biotechnology, Inc., Dallas, TX, USA), p-JNK mouse mAb (0.1% concentration, sc-6254, Santa Cruz Biotechnology, Inc., Dallas, TX, USA), NRF2 rabbit mAb (0.1% concentration, 20733S, CST, Danvers, MA, USA), HO-1 rabbit mAb (diluted 1:1000, Abcam, Cambridge, UK), and GAPDH rabbit mAb (0.01% concentration, ab181602, Abcam, Cambridge, UK). After washing with phosphate-buffered saline with tween-20 (PBST), the membranes were incubated with the corresponding secondary antibody—Goat Anti-Rabbit IgG (H + L) HRP (1:4000 dilution, S0001, Affinity, Shanghai, China) or Goat Anti-Mouse IgG (H + L) HRP (1:4000 dilution, S0002, Affinity, Shanghai, China)—for 1 h at room temperature. Finally, the membranes were treated with enhanced chemiluminescence (ECL) reagent (Thermo Fisher Scientific, Waltham, MA, USA), and signal capture was performed using a bioanalytical imaging system (Clinx, Shanghai, China).

### 4.7. Analytical Statistics

Continuous variables are presented as means ± standard error of the mean (SEM). Composite ordinal variables are presented as median and interquartile range (IQR). Prior to statistical analysis, the normality of data distribution was evaluated using the Shapiro–Wilk test. Differences between groups were analyzed by one-way analysis of variance (ANOVA) for continuous variables with normal distribution, whereas the Kruskal–Wallis test followed by Dunn’s multiple comparisons was used for ordinal variables. Statistical significance was set at *p* < 0.05. All graphs were generated using Prism 8 (GraphPad Software, LLC, San Diego, CA, USA).

Metabolomics data were analyzed using SIMCA 15.1 (Umetrics, Umeå, Sweden) for orthogonal partial least squares-discriminant analysis (OPLS-DA) and model validation. Differential metabolites were identified based on multivariate statistical criteria: variable importance in projection (VIP) > 1, *p* < 0.05, and fold change (FC) ≥ 1.5. Visualization of these metabolites was conducted using R packages (v4.2.2). Additionally, MetaboAnalyst 4.0 (http://www.metaboanalyst.ca/, accessed on 29 December 2024) was utilized to analyze related metabolic pathways.

## 5. Conclusions

In summary, this study demonstrates that XOR inhibition by febuxostat alleviates hepatic steatosis, metabolic dysregulation, and plasma oxidative stress in male MASLD rats, an effect accompanied by alterations in the JNK/NRF2/HO-1 signaling pathway.

## Figures and Tables

**Table 1:** NAFLD activity score grading steatosis, lobular inflammation, and hepatocyte ballooning.

	Control	HF	HF + F
Steatosis	0 (0–0)	3 (2.25–3) *	0.5 (0–1)
Lobular inflammation	0 (0–0.75)	1.5 (1–2) *	1 (1–1.75)
Hepatocyte ballooning	1 (1–1)	2 (2–2) **	1.5 (1–2)
NAS	1 (1–1)	6 (6–6.75) **	3 (3–3.75)

Note: Values are median (IQR). *n* = 3 per group. Statistics: Kruskal–Wallis test with Dunn’s post hoc test for multiple comparisons. * *p* < 0.05, ** *p* < 0.05, compared to control.

## Data Availability

The original contributions presented in this study are included in the article/Supplementary Material. Further inquiries can be directed to the corresponding author.

## Supplementary Materials

The following supporting information can be downloaded at: https://www.mdpi.com/article/10.3390/ijms27021069/s1.

## References

[1] Jamialahmadi O., De Vincentis A., Tavaglione F., Malvestiti F., Li-Gao R., Mancina R.M., Alvarez M., Gelev K., Maurotti S., Vespasiani-Gentilucci U. (2024). Partitioned polygenic risk scores identify distinct types of metabolic dysfunction-associated steatotic liver disease. Nat. Med..

[2] Ha S., Wong V.W., Zhang X., Yu J. (2024). Interplay between gut microbiome, host genetic and epigenetic modifications in MASLD and MASLD-related hepatocellular carcinoma. Gut.

[3] European Association for the Study of the Liver (EASL), European Association for the Study of Diabetes (EASD), European Association for the Study of Obesity (EASO) (2024). EASL-EASD-EASO Clinical Practice Guidelines on the management of metabolic dysfunction-associated steatotic liver disease (MASLD). J. Hepatol..

[4] Younossi Z.M., Kalligeros M., Henry L. (2025). Epidemiology of metabolic dysfunction-associated steatotic liver disease. Clin. Mol. Hepatol..

[5] Mejía-Guzmán J.E., Belmont-Hernández R.A., Chávez-Tapia N.C., Uribe M., Nuño-Lámbarri N. (2025). Metabolic-Dysfunction-Associated Steatotic Liver Disease: Molecular Mechanisms, Clinical Implications, and Emerging Therapeutic Strategies. Int. J. Mol. Sci..

[6] Berlingerio S.P., Bondue T., Tassinari S., Siegerist F., Ferrulli A., Lismont C., Cairoli S., Goffredo B.M., Ghesquière B., Fransen M. (2025). Targeting oxidative stress-induced lipid peroxidation enhances podocyte function in cystinosis. J. Transl. Med..

[7] Del Calvo G., Pollard C.M., Baggio Lopez T., Borges J.I., Suster M.S., Lymperopoulos A. (2024). Nicotine Diminishes Alpha2-Adrenergic Receptor-Dependent Protection Against Oxidative Stress in H9c2 Cardiomyocytes. Drug Des. Dev. Ther..

[8] Wang H., Su S., An X., Xu Y., Sun J., Zhen M., Wang C., Bai C. (2025). A charge reversal nano-assembly prevents hepatic steatosis by resolving inflammation and improving lipid metabolism. Bioact. Mater..

[9] Allameh A., Niayesh-Mehr R., Aliarab A., Sebastiani G., Pantopoulos K. (2023). Oxidative Stress in Liver Pathophysiology and Disease. Antioxidants.

[10] Feng X., Zhang R., Yang Z., Zhang K., Xing J. (2024). Mechanism of Metabolic Dysfunction-associated Steatotic Liver Disease: Important role of lipid metabolism. J. Clin. Transl. Hepatol..

[11] Accacha S., Barillas-Cerritos J., Srivastava A., Ross F., Drewes W., Gulkarov S., De Leon J., Reiss A.B. (2025). From Childhood Obesity to Metabolic Dysfunction-Associated Steatotic Liver Disease (MASLD) and Hyperlipidemia Through Oxidative Stress During Childhood. Metabolites.

[12] Ye S., Hu X., Sun S., Su B., Cai J., Jiang J. (2024). Oridonin promotes RSL3-induced ferroptosis in breast cancer cells by regulating the oxidative stress signaling pathway JNK/Nrf2/HO-1. Eur. J. Pharmacol..

[13] Korsmo H.W., Ekperikpe U.S., Daehn I.S. (2024). Emerging Roles of Xanthine Oxidoreductase in Chronic Kidney Disease. Antioxidants.

[14] Xu C., Wan X., Xu L., Weng H., Yan M., Miao M., Sun Y., Xu G., Dooley S., Li Y. (2015). Xanthine oxidase in non-alcoholic fatty liver disease and hyperuricemia: One stone hits two birds. J. Hepatol..

[15] Tutino V., De Nunzio V., Donghia R., Aloisio Caruso E., Cisternino A.M., Iacovazzi P.A., Mastrosimini A.M., Fernandez E.A., Giannuzzi V., Notarnicola M. (2024). Significant Increase in Oxidative Stress Indices in Erythrocyte Membranes of Obese Patients with Metabolically-Associated Fatty Liver Disease. J. Pers. Med..

[16] Becker M.A., Schumacher H.R., Wortmann R.L., MacDonald P.A., Eustace D., Palo W.A., Streit J., Joseph-Ridge N. (2005). Febuxostat compared with allopurinol in patients with hyperuricemia and gout. N. Engl. J. Med..

[17] Zhao K., Zhang H., Ding W., Yu X., Hou Y., Liu X., Li X., Wang X. (2025). Adipokines regulate the development and progression of MASLD through organellar oxidative stress. Hepatol. Commun..

[18] Zhang S., Zhao R., Wang R., Lu Y., Xu M., Lin X., Lan R., Zhang S., Tang H., Fan Q. (2025). Weissella viridescens Attenuates Hepatic Injury, Oxidative Stress, and Inflammation in a Rat Model of High-Fat Diet-Induced MASLD. Nutrients.

[19] Miao L., Targher G., Byrne C.D., Cao Y.Y., Zheng M.H. (2024). Current status and future trends of the global burden of MASLD. Trends Endocrinol. Metab..

[20] Mikkelsen A.C.D., Kjærgaard K., Schapira A.H.V., Mookerjee R.P., Thomsen K.L. (2025). The liver-brain axis in metabolic dysfunction-associated steatotic liver disease. Lancet Gastroenterol. Hepatol..

[21] Al-Shargi A., El Kholy A.A., Adel A., Hassany M., Shaheen S.M. (2023). Allopurinol versus Febuxostat: A New Approach for the Management of Hepatic Steatosis in Metabolic Dysfunction-Associated Steatotic Liver Disease. Biomedicines.

[22] Kraev K.I., Geneva-Popova M.G., Hristov B.K., Uchikov P.A., Popova-Belova S.D., Kraeva M.I., Basheva-Kraeva Y.M., Stoyanova N.S., Mitkova-Hristova V.T. (2023). Celebrating Versatility: Febuxostat’s Multifaceted Therapeutic Application. Life.

[23] Saito Y., Tanaka A., Yoshida H., Nakashima H., Ban N., Matsuhisa M., Kobayashi Y., Node K. (2024). On Behalf of The Prize Study Investigators. Effects of Xanthine Oxidase Inhibition by Febuxostat on Lipid Profiles of Patients with Hyperuricemia: Insights from Randomized PRIZE Study. Nutrients.

[24] Nishikawa T., Nagata N., Shimakami T., Shirakura T., Matsui C., Ni Y., Zhuge F., Xu L., Chen G., Nagashimada M. (2020). Xanthine oxidase inhibition attenuates insulin resistance and diet-induced steatohepatitis in mice. Sci. Rep..

[25] Yisireyili M., Hayashi M., Wu H., Uchida Y., Yamamoto K., Kikuchi R., Shoaib Hamrah M., Nakayama T., Wu Cheng X., Matsushita T. (2017). Xanthine oxidase inhibition by febuxostat attenuates stress-induced hyperuricemia, glucose dysmetabolism, and prothrombotic state in mice. Sci. Rep..

[26] Yagi H., Akazawa H., Liu Q., Yamamoto K., Nawata K., Saga-Kamo A., Umei M., Kadowaki H., Matsuoka R., Shindo A. (2025). XOR-Derived ROS in Tie2-Lineage Cells Including Endothelial Cells Promotes Aortic Aneurysm Progression in Marfan Syndrome. Arterioscler. Thromb. Vasc. Biol..

[27] Wang L., Jiang L., Chu Y., Feng F., Tang W., Chen C., Qiu Y., Hu Z., Diao H., Tang Z. (2023). Dietary Taurine Improves Growth Performance and Intestine Health via the GSH/GSSG Antioxidant System and Nrf2/ARE Signaling Pathway in Weaned Piglets. Antioxidants.

[28] Aruoma O.I., Halliwell B., Hoey B.M., Butler J. (1988). The antioxidant action of taurine, hypotaurine and their metabolic precursors. Biochem. J..

[29] Nakamura H., Yatsuki J., Ubuka T. (2006). Production of hypotaurine, taurine and sulfate in rats and mice injected with L-cysteinesulfinate. Amino Acids.

[30] Zhu W., Li G., Shi H., Ruan Y., Liu C. (2024). Transcriptome and Metabolome Analyses Reveal the Regulatory Mechanism of TC1a in the Sucrose and Starch Synthesis Pathways in *Arabidopsis thaliana*. Plants.

[31] Manna P., Achari A.E., Jain S.K. (2017). Vitamin D supplementation inhibits oxidative stress and upregulate SIRT1/AMPK/GLUT4 cascade in high glucose-treated 3T3L1 adipocytes and in adipose tissue of high fat diet-fed diabetic mice. Arch. Biochem. Biophys..

[32] Deja S., Crawford P.A., Burgess S.C. (2022). Krebs takes a turn at cell differentiation. Cell Metab..

[33] Strijbis K., van Roermund C.W., van den Burg J., van den Berg M., Hardy G.P., Wanders R.J., Distel B. (2010). Contributions of carnitine acetyltransferases to intracellular acetyl unit transport in Candida albicans. J. Biol. Chem..

[34] Wu B., Woo J.S., Hasiakos S., Pan C., Cokus S., Benincá C., Stiles L., Sun Z., Pellegrini M., Shirihai O.S. (2025). Mitochondrial reactive oxygen species regulate acetyl-CoA flux between cytokine production and fatty acid synthesis in effector T cells. Cell Rep..

[35] Cao H., Cai Q., Guo W., Su Q., Qin H., Wang T., Xian Y., Zeng L., Cai M., Guan H. (2023). Malonylation of Acetyl-CoA carboxylase 1 promotes hepatic steatosis and is attenuated by ketogenic diet in NAFLD. Cell Rep..

[36] Luo C., Lian X., Hong L., Zou J., Li Z., Zhu Y., Huang T., Zhang Y., Hu Y., Yuan H. (2016). High Uric Acid Activates the ROS-AMPK Pathway, Impairs CD68 Expression and Inhibits OxLDL-Induced Foam-Cell Formation in a Human Monocytic Cell Line, THP-1. Cell. Physiol. Biochem..

[37] Gupta N., Singh K. (2025). Exploring the role of xanthine oxidase and aldehyde oxidase in metabolic dysfunction-associated steatotic liver disease (MASLD). J. Mol. Histol..

[38] Bortolotti M., Polito L., Battelli M.G., Bolognesi A. (2021). Xanthine oxidoreductase: One enzyme for multiple physiological tasks. Redox Biol..

[39] Pinz M.P., Medeiros I., Carvalho L.A.D.C., Meotti F.C. (2025). Is uric acid a true antioxidant? Identification of uric acid oxidation products and their biological effects. Redox Rep..

[40] Wang X.L., Li L., Meng X. (2024). Interplay between the Redox System and Renal Tubular Transport. Antioxidants.

[41] Zeng N., Wu F., Lu J., Li X., Lin S., Zhou L., Wang Z., Wu G., Huang Q., Zheng D. (2024). High-fat diet impairs gut barrier through intestinal microbiota-derived reactive oxygen species. Sci. China Life Sci..

[42] Li H., Li X., Wang Y., Han W., Li H., Zhang Q. (2024). Hypoxia-Mediated Upregulation of Xanthine Oxidoreductase Causes DNA Damage of Colonic Epithelial Cells in Colitis. Inflammation.

[43] Esteras N., Abramov A.Y. (2022). Nrf2 as a regulator of mitochondrial function: Energy metabolism and beyond. Free Radic. Biol. Med..

[44] Jin X., Xu Z., Cao J., Yan R., Xu R., Ran R., Ma Y., Cai W., Fan R., Zhang Y. (2017). HO-1/EBP interaction alleviates cholesterol-induced hypoxia through the activation of the AKT and Nrf2/mTOR pathways and inhibition of carbohydrate metabolism in cardiomyocytes. Int. J. Mol. Med..

[45] Liao W., Yang W., Shen Z., Ai W., Pan Q., Sun Y., Guo S. (2021). Heme Oxygenase-1 Regulates Ferrous Iron and Foxo1 in Control of Hepatic Gluconeogenesis. Diabetes.

[46] Esteras N., Dinkova-Kostova A.T., Abramov A.Y. (2016). Nrf2 activation in the treatment of neurodegenerative diseases: A focus on its role in mitochondrial bioenergetics and function. Biol. Chem..

[47] Seidel U., Huebbe P., Rimbach G. (2019). Taurine: A Regulator of Cellular Redox Homeostasis and Skeletal Muscle Function. Mol. Nutr. Food Res..

[48] Dumitru A., Tocia C., Bădescu A.C., Trandafir A., Alexandrescu L., Popescu R., Dumitru E., Chisoi A., Manea M., Matei E. (2025). Linking gut permeability to liver steatosis: Noninvasive biomarker evaluation in MASLD patients—A prospective cross-sectional study. Medicine.

[49] Abu-Risha S.E., El-Mahdy N.A., El-Hosiny F.T., Elhusseiny M.E., El-Kadem A.H. (2025). Anticolitis effect of febuxostat. The imperative role of NLRP-3/Caspase-1/IL-1β pathway via histopathological, immunohistochemical and biochemical approach. Immunopharmacol. Immunotoxicol..

[50] Booth F.W., Roberts C.K., Laye M.J. (2012). Lack of exercise is a major cause of chronic diseases. Compr. Physiol..

[51] Kanemitsu T., Tsurudome Y., Kusunose N., Oda M., Matsunaga N., Koyanagi S., Ohdo S. (2017). Periodic variation in bile acids controls circadian changes in uric acid via regulation of xanthine oxidase by the orphan nuclear receptor PPARα. J. Biol. Chem..

[52] Meyer J., Teixeira A.M., Richter S., Larner D.P., Syed A., Klöting N., Matz-Soja M., Gaul S., Barnikol-Oettler A., Kiess W. (2025). Sex differences in diet-induced MASLD–are female mice naturally protected?. Front. Endocrinol..

[53] Basil B., Myke-Mbata B.K., Eze O.E., Akubue A.U. (2024). From adiposity to steatosis: Metabolic dysfunction-associated steatotic liver disease, a hepatic expression of metabolic syndrome—Current insights and future directions. Clin. Diabetes Endocrinol..

[54] Lv Y., Sun M., He Y., Zhang X., Min Y., Liu L., Yu W. (2025). Effects of induced molting on lipid accumulation in liver of aged laying hens. Poult. Sci..

[55] Xia Y., Li L., Li D., Liu Y., Hao L. (2025). Serum Metabolomic Analysis of Healthy and Central Precocious Puberty Girls. Clin. Endocrinol..

[56] Yu Y., Liu P., Liu Y., Zhang Z., Kong X., Wang N., Sun X., Xu C., Bai X., Su W. (2025). Discovery of metabolic biomarkers for distinguishing LAA and SVO subtypes of acute ischemic stroke. Sci. Rep..

