# Supplementary material for: Febuxostat Improves MASLD in Male Rats: Roles of XOR Inhibition and Associated JNK/NRF2/HO-1 Pathway Changes
Source: Int J Mol Sci. 2026 Jan 21;27(2):1069. doi: 10.3390/ijms27021069 (PMC12841615; doi:10.3390/ijms27021069)
Supplement: Supplementary file 1 [file ijms-27-01069-s001.zip › ijms-3994777-supplementary.pdf]

Supplementary 1:

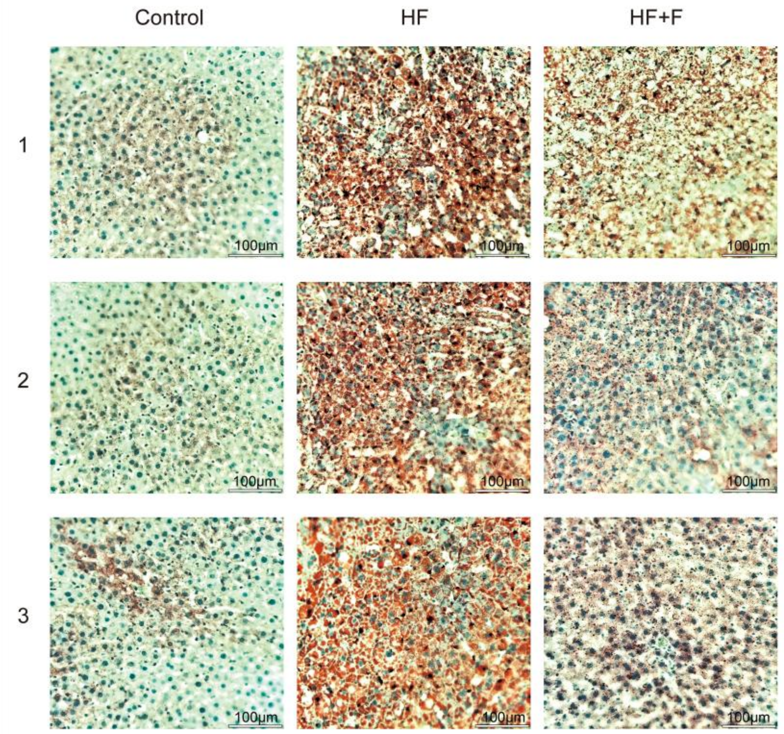

Figure S1. All the images of Oil Red O staining. *n* = 3 per group.

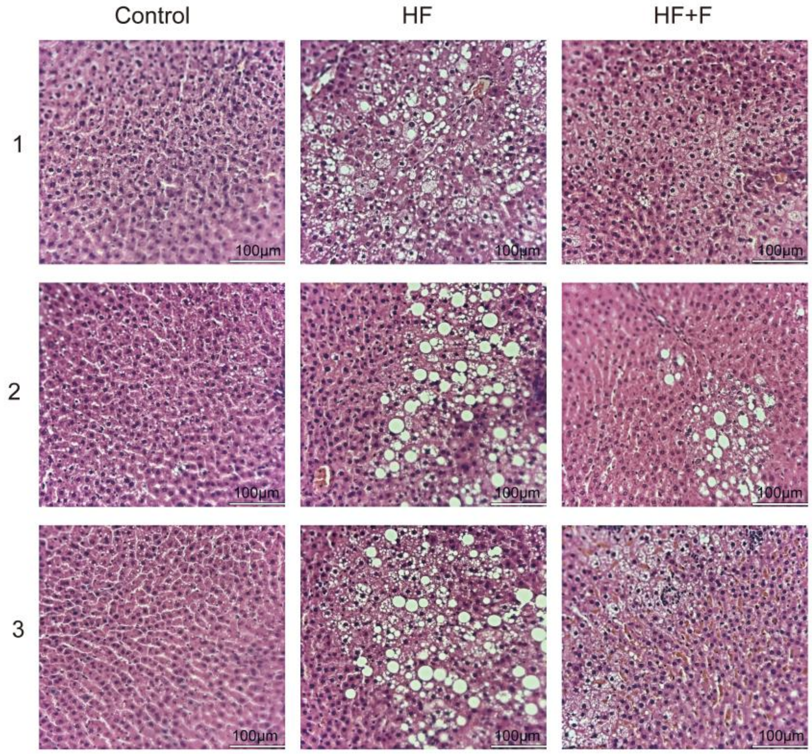

Figure S2. All the images of Hematoxylin and eosin staining. *n* = 3 per group.

Supplementary 2:

Table S1. Liver coefficient and epididymal fat coefficient.

	Control	HF	HF+F
Liver coefficient	3.41%±0.20%	3.97%±0.45%	3.57%±0.26%
Epididymal fat coefficient	2.93%±0.17%	3.38%±0.79%	3.17%±0.24%

Note: Liver coefficient [calculated as (liver weight / body weight) × 100%] and epididymal fat coefficient [calculated as (epididymal fat weight / body weight) × 100%] in different groups (n = 6 per group). All data are expressed as mean ± SEM.

Supplementary 3:

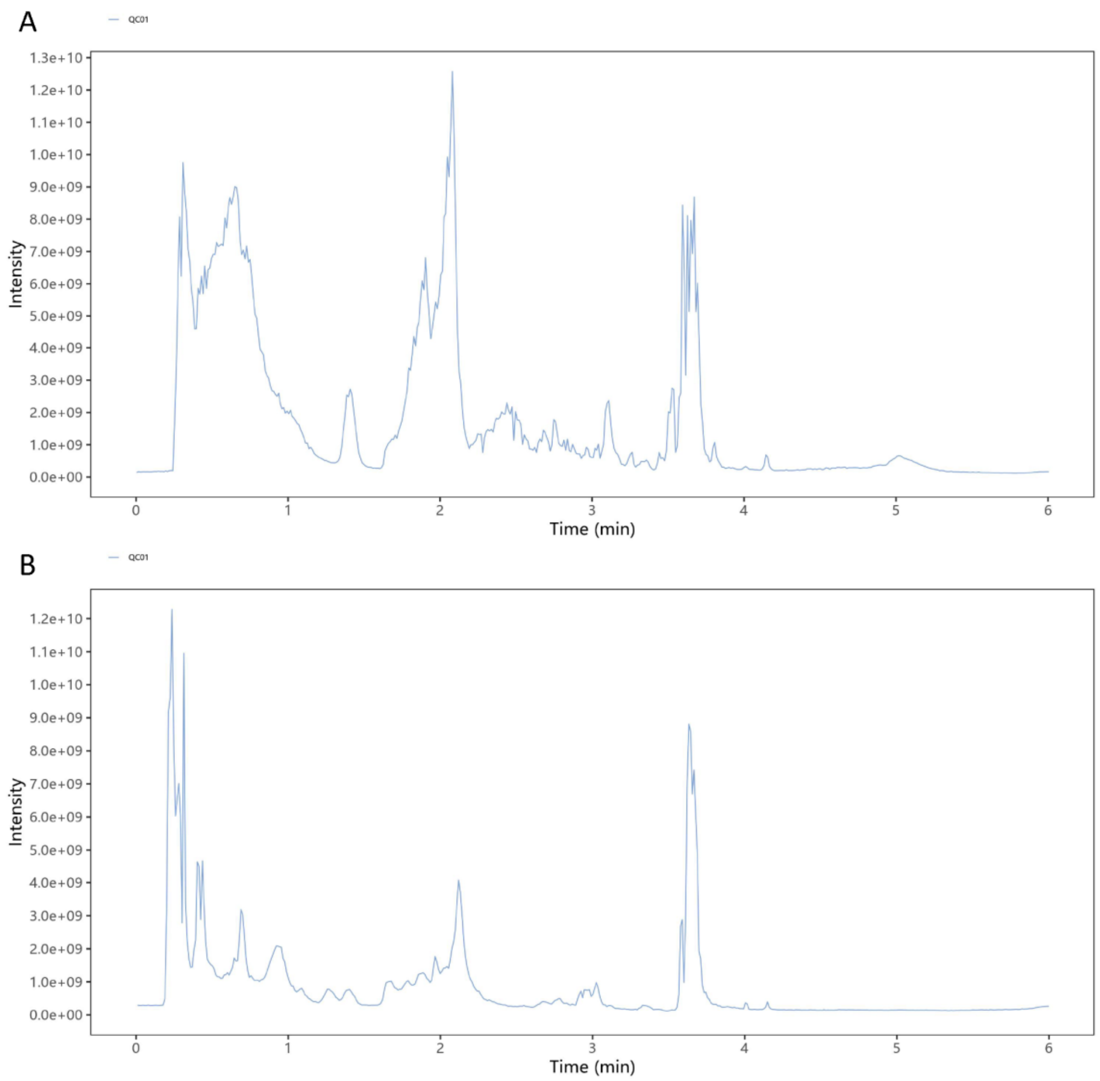

Figure S3. Total Ion Chromatograms of Serum Quality Control Samples in Positive (A) and Negative (B) Ion Modes by UHPLC-QE-MS

Supplementary 4:

Table S2. List of the 51 differentially abundant metabolites identified in the metabolomic analysis.

Compounds	*m/z*	RT	VIP	*p* value	FC	Log_2FC
N~1~-Methyl-4-nitro-1,2-benzenediamine	168.08	195.10	1.00	0.02	2.74	1.45
2-Cyano-N-(3,4-difluorophenyl)acetamide	197.05	187.80	1.06	0.04	2.62	1.39
Nomifensine	239.15	30.30	1.06	0.03	1.62	0.70
Pentaethylene glycol	239.15	30.30	1.06	0.03	1.62	0.70
Thymine	125.04	32.00	1.07	0.05	1.82	0.87
1H-Indole-3-carboxamide, 1-(5-fluoropentyl)-N-2-naphthalenyl-	375.19	203.00	1.10	0.04	0.49	-1.04
3-Sulfinoalanine	152.00	94.80	1.12	0.01	2.17	1.12
1,5-Anhydroglucitol	163.06	116.60	1.14	0.03	0.54	-0.89
cis-11-Eicosenoic acid	309.28	21.10	1.14	0.01	0.61	-0.70
Hydroxyphenyllactic acid	181.05	105.80	1.15	0.03	1.57	0.65
4-Oxohexanoic acid	129.06	52.60	1.16	0.02	0.49	-1.04

1-Stearoyl-2-arachidonoyl-sn-glycero-3-phosphoserine	810.53	127.00	1.18	0.05	2.13	1.09
7-ethenyl-1,4a,7-trimethyl-3,4,6,8,8a,9,10,10a-octahydro-2H-phenanthrene-1-carboxylic acid	303.23	25.40	1.20	0.04	0.41	-1.29
N5-(1-Iminoethyl)-L-ornithine	174.12	307.30	1.23	0.03	1.97	0.98
Propanedinitrile, 2-[(3,4,5-trihydroxyphenyl)methylene]-	201.03	116.40	1.24	0.03	0.57	-0.81
1-(2-Hydroxyethyl)pyrazole	113.07	230.50	1.24	0.02	1.63	0.71
D-Ribose	149.05	95.40	1.26	0.01	1.96	0.97
3-Hydroxy-3-(methoxycarbonyl)pentanedioic acid	205.03	27.00	1.26	0.05	3.09	1.63
Hexaethylene glycol	283.18	32.20	1.27	0.02	1.66	0.73
2,4,6-Trimethylpyridine	122.10	16.80	1.27	0.02	1.96	0.97
cis-11.14-Eicosadienoic acid	307.26	21.50	1.27	0.03	0.38	-1.40
(Dimethoxymethyl)benzene	153.09	199.40	1.27	0.02	1.96	0.97
3-Hydroxy-3H-benzo[de]isochromen-1-one	199.04	116.10	1.30	0.04	0.57	-0.82
beta-Alaninamide	71.06	173.60	1.30	0.01	0.62	-0.69

N-Acetyl-asparagine	173.06	188.40	1.30	0.00	2.38	1.25
1,2-Distearoyl-sn-glycero-3-phospho-L-serine	790.56	45.70	1.33	0.01	0.48	-1.05
Pro-Gly	173.09	199.60	1.33	0.05	2.07	1.05
Anacardic acid	347.26	25.00	1.33	0.01	0.23	-2.15
Fenoldopam	304.07	181.80	1.33	0.05	2.28	1.19
6-Nitro-1,2,3-benzotriazin-4(3H)-one	191.02	99.20	1.35	0.03	2.71	1.44
Glucose	179.06	184.30	1.36	0.04	2.13	1.09
Mannose	179.06	184.30	1.36	0.04	2.13	1.09
L-Erythrulose	119.03	184.30	1.37	0.03	2.13	1.09
2-(1-Hydroxycyclohexyl)butanoic acid	185.12	29.30	1.38	0.04	0.42	-1.23
Malic acid	133.01	228.40	1.38	0.04	2.09	1.06
4-Ethoxy-4-oxobut-2-enoic acid	143.03	184.50	1.39	0.05	1.82	0.87
2-Hydroxyhexanedioic acid	143.03	203.90	1.41	0.04	1.97	0.98

1H-Indazole-3-carboxamide, N-[(1S)-1-(aminocarbonyl)-2-methylpropyl]-1-pentyl-	331.21	205.70	1.42	0.03	2.11	1.08
cis-7-Hexadecenoic acid	253.22	23.80	1.43	0.02	0.57	-0.80
LPG(18:1(9Z))	509.29	59.70	1.43	0.04	0.59	-0.75
2-((2R)-2-Hydroxycyclohexyl)acetic acid	157.09	36.80	1.44	0.01	0.37	-1.44
Dye X-27237-115	328.04	147.50	1.44	0.01	0.58	-0.80
cis-Pinonic acid	183.10	33.00	1.49	0.02	0.46	-1.12
4-Chlorophenylboronic acid	155.01	133.00	1.50	0.00	0.54	-0.88
3,5,9-Trioxa-4-phosphatetracosan-1-aminium, 7-(acetyloxy)-24-carboxy-4-hydroxy-N,N,N-trimethyl-, inner salt, 4-oxide, (R)-	552.33	134.20	1.55	0.04	0.39	-1.35
6-Oxocativic acid	319.23	25.20	1.56	0.03	0.28	-1.84
1-Piperazineacetic acid, 4-(phenylmethyl)-, 2-[[2-hydroxy-3-(2-propen-1-yl)phenyl]methylene]hydrazide	393.22	100.10	1.71	0.02	0.31	-1.67
8-Hydroxy-3-(7-hydroxyheptyl)-6-oxo-3,4-dihydroisochromene-7-carboxylic acid	321.14	94.70	1.73	0.02	1.60	0.68
Dihydrokaempferol	287.05	183.50	1.74	0.05	0.67	-0.59
(2,2,3,3-Tetrafluoropropoxy)acetic acid	189.01	229.30	1.84	0.00	0.65	-0.61

N-Acetylmethionine	190.05	116.10	1.87	0.01	0.55	-0.86

m/z: mass-to-charge ratio; RT: retention time (min); VIP: variable importance in projection; *p* value: statistical significance; FC: fold change; *p* value calculated by student's T test.

Supplementary 5: Composition of the high-fat diet and normal diet

Normal diet: A standard rodent chow (Xietong Pharmaceutical Science and Engineering Co., Ltd., Beijing, China) — with an energy distribution of 23.1% of total energy from protein, 64.3% from carbohydrates, and 12.7% from fat — was formulated with Northeast China corn, wheat, chicken meal, and other conventional feed ingredients.

High-fat diet: A purified high-fat rodent chow (MD12033, Medicience Ltd, Jiangsu, China) — with an energy distribution of 20.0% of total energy from protein, 20.0% from carbohydrates, and 60.0% from fat — was formulated with lard, vegetable oil, maltodextrin, and other auxiliary ingredients.
